# Supplementary material for: Detection of Leishmania spp. in Chronic Dermatitis: Retrospective Study in Exposed Horse Populations
Source: Pathogens. 2022 May 31;11(6):634. doi: 10.3390/pathogens11060634 (PMC9227255; doi:10.3390/pathogens11060634)
Supplement: Supplementary file 1 [file pathogens-11-00634-s001.zip › pathogens-1716355-supplementary.pdf]

Supplementary Table S1. Severity, pattern and distribution of the inflammatory infiltrate in the samples. Case ID. 18(*) was the positive sample.

Case Number (ID)	Severity	Pattern	Distribution
1	moderate	nodular	deep
2	moderate	interstitial	deep
3	mild	perivascular	superficial
4	moderate	interstitial	deep
5	moderate	interstitial	mid
6	moderate	interstitial	mid
7	moderate	perivascular	superficial
8	severe	nodular	deep
9	moderate	nodular	deep
10	severe	nodular	deep
11	mild	perivascular	superficial
12	mild	perivascular	superficial
13	severe	nodular	deep
14	mild	perivascular	superficial
15	moderate	interstitial	deep
16	moderate	interstitial	mid
17	moderate	perivascular	mid
**18***	**severe**	**nodular**	**deep**
19	mild	perivascular	mid
20	mild	perivascular	deep
21	moderate	interstitial	mid
22	severe	nodular	superficial
23	mild	perivascular	deep
24	severe	nodular	deep
25	mild	perivascular	superficial
26	moderate	nodular	deep
27	moderate	nodular	deep
28	moderate	interstitial	deep
29	severe	interstitial	superficial
30	severe	interstitial	deep
31	moderate	nodular	deep
32	moderate	interstitial	deep
33	severe	nodular	deep
34	moderate	perivascular	mid
35	mild	perivascular	superficial
36	moderate	interstitial	deep
37	moderate	interstitial	superficial
38	moderate	perivascular	superficial
39	moderate	diffuse	mid
40	moderate	nodular	deep
41	moderate	interstitial	mid
42	severe	diffuse	deep
43	mild	perivascular	superficial
44	moderate	interstitial	deep
45	severe	diffuse	deep
46	moderate	diffuse	deep
47	mild	perivascular	superficial
